# Angiosarcoma Arising in Ovarian Mucinous Tumor: A Challenge in Intraoperative Frozen Section Diagnosis

**DOI:** 10.1155/2016/8508624

**Published:** 2016-10-31

**Authors:** Surapan Khunamornpong, Jongkolnee Settakorn, Kornkanok Sukpan, Tip Pongsuvareeyakul, Sumalee Siriaunkgul

**Affiliations:** Department of Pathology, Faculty of Medicine, Chiang Mai University, Chiang Mai 50200, Thailand

## Abstract

Angiosarcoma of the ovary is rare but represents an aggressive type of malignant ovarian neoplasms. The purpose of this report is to describe the features of angiosarcoma arising in mucinous tumor that was misinterpreted as a benign vascular proliferation during the intraoperative consultation. A 45-year-old woman presented with an abdominal mass for 1 month. Exploratory laparotomy was performed. A 35 cm right ovarian mass submitted for intraoperative consultation was a multicystic mucinous tumor with an 8 cm area of hemorrhagic lesion between cystic locules. The frozen section diagnosis was at least mucinous borderline tumor. The hemorrhagic area, which was intraoperatively interpreted as organizing vessels associated with previous hemorrhage, represented angiosarcoma in permanent sections. Angiosarcoma may present a challenge in intraoperative frozen section diagnosis of an ovarian mass. The presence of ectatic anastomosing vessels with dissecting growth appears to be the clue to a suspicion of angiosarcoma. The presence of endothelial atypia provides further support for the diagnosis. A macroscopic hemorrhagic area in an ovarian mucinous tumor should be evaluated with care, and the possibility of angiosarcoma should be borne in mind.

## 1. Introduction

Mucinous tumor is a common type of ovarian epithelial tumors. The large majority of mucinous tumors are benign and borderline tumors. Invasive mucinous adenocarcinoma is uncommon and usually arises in the background of benign or borderline tumors. Nonepithelial malignant neoplasms sometimes arise in mucinous tumors, typically as mural nodules.

Angiosarcoma of the ovary is rare but represents an aggressive type of malignant ovarian neoplasms. To our knowledge, less than 40 cases of ovarian angiosarcoma have been reported in English literature [1–3]. In two-thirds of cases, ovarian angiosarcomas were described in a pure form [3]. In the remaining cases, angiosarcoma was associated with ovarian teratoma or epithelial neoplasm [2, 4]. Only 5 previous cases of angiosarcoma related to mucinous tumors have been reported [1, 2, 5–7].

Due to the rarity of angiosarcoma in the ovary, this tumor can be a diagnostic challenge to surgical pathologists, particularly during an intraoperative consultation. A failure to intraoperatively recognize this malignant neoplasm may result in incomplete surgical staging procedure or inappropriate clinical management. The purpose of this report was to describe the features of angiosarcoma arising in mucinous tumor that was initially misinterpreted as a nonneoplastic vascular proliferation in the intraoperative frozen section examination. The findings in this case may be useful for pathologists in the recognition of this uncommon type of ovarian tumors.

## 2. Case Presentation

A 45-year-old woman, parity 0, presented with a complaint of a palpable abdominal mass with discomfort for 1 month. The physical examination showed unremarkable finding except for a large pelvic mass. The serologic tumor markers were as follows: CA 125, 110 IU/mL (normal < 35), CA 19-9, 144 IU/mL (normal < 35), and CEA, 1.3 *μ*g/L (normal < 3.4). Ultrasonography and abdominal computed tomographic scan revealed a 26 cm right ovarian solid-cystic mass with internal septation. No abnormality in the other intra-abdominal organs was identified. Exploratory laparotomy was performed. Intraoperatively, there was 200 mL of mucous fluid. A 35 cm right ovarian mass was identified, with focal previous rupture of the external surface. The left ovary showed a 6 cm cyst with chocolate-like content. The other gynecologic and intra-abdominal organs were unremarkable.

**Figure 1:**
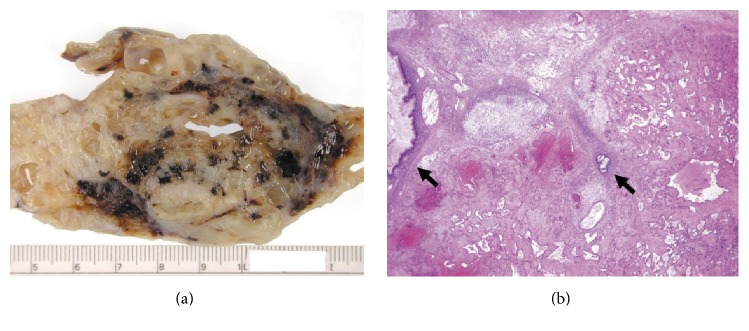
Macroscopic and intraoperative histologic appearance of ovarian tumor. (a) A confluence of hemorrhagic foci in spongy to solid portion of ovarian cystic mass. (b) Ectatic anastomosing vascular spaces in the frozen section sample; arrows indicating mucinous cyst lining (defrosted tissue; hematoxylin and eosin [H&E] stain: ×40).

The right ovarian mass was submitted for intraoperative consultation. The sectioned surface of the mass was multicystic and composed of numerous mucus-filled cystic locules and spongy to almost solid tissue. An area of hemorrhagic foci between cystic and spongy locules is noted, with a maximal linear dimension of 8 cm (Figure 1(a)). The frozen section diagnosis was at least mucinous borderline tumor, pending adequate tissue sampling for permanent sections to rule out mucinous adenocarcinoma. The hemorrhagic area sampled in the frozen section was interpreted as organizing vessels associated with intratumoral hemorrhage (Figure 1(b)). The patient underwent a complete surgical staging procedure including hysterectomy with bilateral salpingooophorectomy, omentectomy, pelvic and para-aortic lymph node dissection, and appendectomy. Postoperatively, the patient received adjuvant chemotherapy for FIGO stage IC ovarian cancer using doxorubicin and ifosfamide. She was lost to follow-up after the administration of one cycle of chemotherapy.

**Figure 2:**
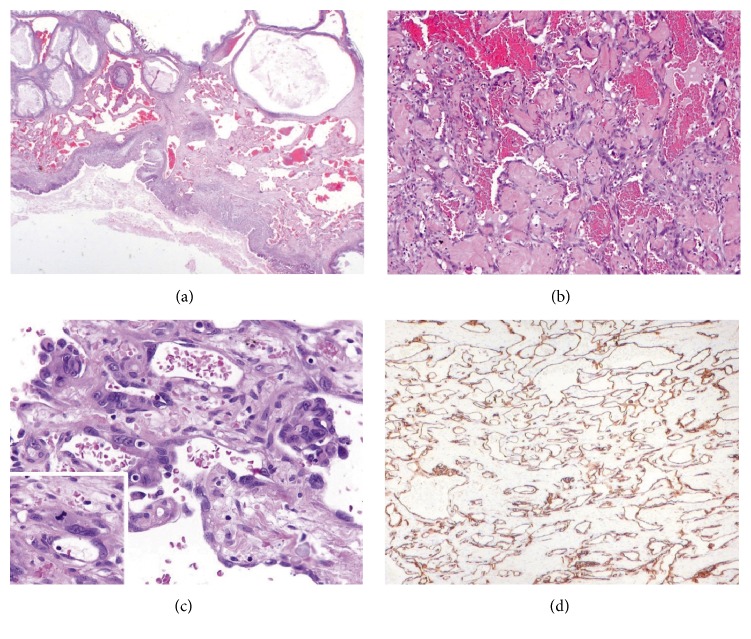
Angiosarcoma in mucinous tumor. (a) Dissecting growth of ectatic vascular channels in a septum between mucinous borderline locules (H&E stain: ×10). (b) Endothelial lining with marked nuclear atypia (H&E stain: ×100). (c) Focal endothelial tufting and mitotic activity (inset) (H&E stain: ×400). (d) Diffuse immunoexpression of CD31 (CD31 immunostain: ×40).

The right ovarian mass was further sampled in 35 tissue blocks for permanent sections. The tumor represented a mucinous borderline tumor with marked nuclear atypia (intraepithelial carcinoma) and few foci of infiltrative-type stromal invasion of less than 5 mm in diameter (microinvasive mucinous adenocarcinoma). In the hemorrhagic area, proliferation of irregular and dilated anastomosing vascular channels was present around small collections of blood and dissected into the septa between mucinous borderline locules (Figure 2). The lining cells of these vascular spaces showed variable degree of nuclear atypia. Endothelial cells with large hyperchromatic nuclei were occasionally observed and focally formed intraluminal glomerulus-like clusters, although these markedly atypical areas were not visualized in the frozen sections. Mitotic figures were scattered, and the maximal mitotic count was 3 in 10 high-power fields. The intervening stroma between vascular spaces was hyalinized in many areas.

**Figure 3:**
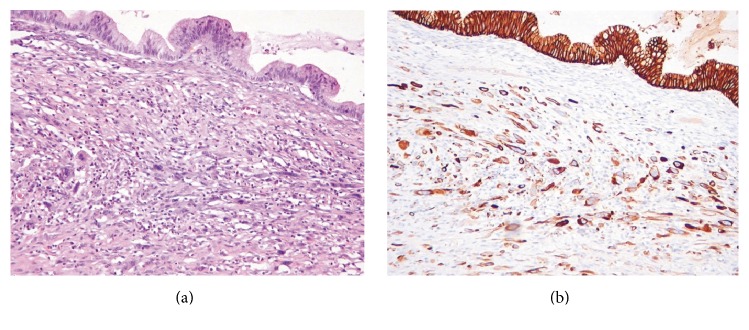
An undifferentiated carcinoma focus in mucinous tumor. (a) An infiltration of pleomorphic spindle-shaped cells beneath a mucinous borderline lining (H&E stain: ×100). (b) Diffuse immunoexpression of cytokeratin (cytokeratin [AE1/AE3] immunostain: ×100).

Another focus of infiltrating undifferentiated carcinoma composed of high-grade pleomorphic spindle and epithelioid cells, measuring 14.0 × 2.5 mm, was present in the wall of a mucinous borderline locule remote to the hemorrhagic area (Figure 3). No connection between angiosarcomatous and the undifferentiated carcinomatous component was identified.

The left ovary contained a 6.5 cm endometriotic cyst. The uterus and bilateral fallopian tubes were not remarkable. The omentum, pelvic and para-aortic lymph node specimens, and appendix were negative for neoplastic involvement.

The immunohistochemical stains showed different staining patterns between angiosarcoma and undifferentiated carcinoma (angiosarcoma: cytokeratin [AE1/AE3]-negative/CD31 and CD34-positive; carcinoma: cytokeratin-positive/CD31 and CD34-negative).

## 3. Discussion

Ovarian angiosarcoma is an aggressive malignant neoplasm. Approximately two-thirds of cases had stage II–IV tumors, and approximately one-half of patients with known follow-up data died of disease with a median survival of 10.2 months [8]. Metastatic lesion may rarely be the presentation without a clinical symptom of ovarian mass [9]. Despite a poor prognosis in many cases, some patients have been reported to achieve a remission of the disease using chemotherapy [1]. Thus, it is of paramount importance that angiosarcoma be detected for proper therapeutic approach and prognostic evaluation.

To our knowledge, 37 cases of ovarian angiosarcoma have been previously reported (mean age 36 years, range 11 to 81 years) [1, 2]. Seven of these cases were associated with ovarian teratomatous origin [4, 10]. Six cases of ovarian angiosarcoma were associated with ovarian epithelial neoplasms: one with serous borderline tumor (age 45 years) [11] and the other 5 cases with mucinous tumors (age 29 to 77 years) [2]. Of these 5 cases, 3 had mucinous cystadenoma (age 29, 39, and 77 years) [1, 5, 7] and 2 had microinvasive or invasive mucinous adenocarcinoma (age 37 and 54 years) [2, 6]. The patients who had angiosarcoma associated with ovarian epithelial tumors had an older mean age than those with pure ovarian angiosarcoma or angiosarcoma associated with teratoma (47 years versus 36 years).

Macroscopic appearance of angiosarcomatous component is typically a hemorrhagic lesion. In 5 previously reported cases of angiosarcoma associated with mucinous tumors, the angiosarcomatous component was macroscopically recognized as mural nodules or hematoma-like areas in 4 cases [1, 2, 6, 7] and as spongy tissue between cystic septa in the remaining case [5]. The macroscopic finding in the latter case is rather similar to the finding in the present case. Such dissecting appearance is difficult to be recognized as another neoplastic component, and it can be easily confused with hemorrhagic areas related to infarction which are occasionally seen in mucinous tumors. Among the cases with nodule-like lesions, angiosarcoma coexisted with granulation tissue nodules in one patient [7]. The angiosarcomatous component in that patient was missed in the initial sampling for histologic examination but was only identified in the repeat sampling after a rapidly fatal clinical course of the patient [7]. This information emphasizes the importance of proper tissue sampling in the detection of angiosarcomatous component in mucinous tumor.

It should be noted that benign vascular neoplasm arising in ovarian mucinous tumor is less common than angiosarcoma. Among approximately 50 reported cases of ovarian hemangioma, only 2 cases of hemangioma were associated with mucinous tumors: one in mucinous cystadenoma (32-year-old) [12] and the other in mucinous borderline tumor with microinvasion (39-year-old) [13]. Thus, a macroscopically recognized hemorrhagic lesion composed of vascular proliferation in ovarian mucinous tumor should raise a concern and be carefully examined to rule out angiosarcoma.

The histologic recognition of angiosarcomatous component in the frozen section of ovarian mass can be difficult [3]. In the report by Yaqoob et al. [3], the intraoperative frozen section diagnosis of angiosarcoma was reported as indeterminate between benign and malignant lesions, and a complete surgical staging was not performed. In the present case, the angiosarcomatous component was included in the frozen section samples but was intraoperatively interpreted as a benign vascular proliferation associated with hemorrhage. The decreased nuclear details and nuclear artifact in frozen sections also contribute to the difficulty in the recognition of endothelial atypia. Thus, it is important that the presence of irregular dilated and anastomosing vascular channels should be a clue for a suspicion of angiosarcoma. Then, endothelial atypia should be carefully searched and, if identified, could provide additional support for an intraoperative diagnosis of angiosarcoma.

Differential diagnosis of angiosarcoma includes benign vascular proliferation or hemangioma. Anastomosing network of vascular channels with intervening hyalinized stroma may be similarly observed in benign papillary endothelial hyperplasia following organization of hematoma or thrombus [13], but this lesion is usually well-demarcated in contrast with angiosarcoma. Benign vascular proliferation can be associated with ovarian teratoma, particularly those containing glial tissue. This vascular proliferation is composed of small vessels resembling capillary hemangioma rather than the ectatic anastomosing vessels in angiosarcoma [14]. Mitotic count is variable in angiosarcoma and increased mitotic activity alone is not diagnostic of malignancy as it may be present in mitotically active hemangioma [10].

Intraoperative frozen section is important in making decision for surgical management, that is, radical resection with complete staging in malignant ovarian tumors. Sampling error is a major cause of inaccurate frozen section diagnoses, and this type of error occurs more commonly in tumors with high tissue heterogeneity, particularly mucinous tumors [15]. As observed in the present case, even a hemorrhagic area within ovarian mucinous tumor should also be sampled for frozen section examination.

## 4. Conclusion 

Angiosarcoma may present a challenge in intraoperative frozen section diagnosis of an ovarian mass. A macroscopic hemorrhagic area in an ovarian mucinous tumor should be evaluated with care, and the possibility of angiosarcoma should be borne in mind. The presence of ectatic anastomosing vessels with dissecting growth appears to be the clue to a suspicion of angiosarcoma. The presence of endothelial atypia provides further support for the diagnosis.
